# Individual Differences in the Post-Illumination Pupil Response to Blue Light: Assessment without Mydriatics

**DOI:** 10.3390/biology5030034

**Published:** 2016-09-09

**Authors:** Jessica Bruijel, Wisse P. van der Meijden, Denise Bijlenga, Farangis Dorani, Joris E. Coppens, Bart H. W. te Lindert, J. J. Sandra Kooij, Eus J. W. Van Someren

**Affiliations:** 1 Department of Sleep and Cognition, Netherlands Institute for Neuroscience, Amsterdam 1105 BA, The Netherlands; jessica_bruyel@hotmail.com (J.B.); j.coppens@nin.knaw.nl (J.E.C.); b.te.lindert@nin.knaw.nl (B.H.W.t.L.); e.van.someren@nin.knaw.nl (E.J.W.V.S.); 2 Sleep Disorders Center, CHU and FMTS, CNRS-UPR 3212, Institute of Cellular and Integrative Neurosciences, University of Strasbourg, Strasbourg 67084, France; 3 Center for Chronobiology, Psychiatric Hospital of the University of Basel, Basel CH-4012, Switzerland; 4 PsyQ Psycho-Medical Programs, Expertise Center Adult ADHD, The Hague 2593 HR, The Netherlands; D.Bijlenga@psyq.nl (D.B.); F.Dorani@psyq.nl (F.D.); s.kooij@psyq.nl (J.J.S.K.); 5 Departments of Integrative Neurophysiology and Psychiatry GGZ InGeest, Center for Neurogenomics and Cognitive Research (CNCR), Neuroscience Campus Amsterdam, VU University and Medical Center, Amsterdam 1081 HV, The Netherlands

**Keywords:** pupil, intrinsically photosensitive retinal ganglion cells, melanopsin, pupil dilation, post-illumination pupil response, light

## Abstract

Melanopsin-containing retinal ganglion cells play an important role in the non-image forming effects of light, through their direct projections on brain circuits involved in circadian rhythms, mood and alertness. Individual differences in the functionality of the melanopsin-signaling circuitry can be reliably quantified using the maximum post-illumination pupil response (PIPR) after blue light. Previous protocols for acquiring PIPR relied on the use of mydriatics to dilate the light-exposed eye. However, pharmacological pupil dilation is uncomfortable for the participants and requires ophthalmological expertise. Hence, we here investigated whether an individual’s maximum PIPR can be validly obtained in a protocol that does not use mydriatics but rather increases the intensity of the light stimulus. In 18 participants (5 males, mean age ± SD: 34.6 ± 13.6 years) we evaluated the PIPR after exposure to intensified blue light (550 µW/cm^2^) provided to an undilated dynamic pupil. The test-retest reliability of the primary PIPR outcome parameter was very high, both between day-to-day assessments (Intraclass Correlation Coefficient (ICC) = 0.85), as well as between winter and summer assessments (ICC = 0.83). Compared to the PIPR obtained with the use of mydriatics and 160 µW/cm^2^ blue light exposure, the method with intensified light without mydriatics showed almost zero bias according to Bland-Altman plots and had moderate to strong reliability (ICC = 0.67). In conclusion, for PIPR assessments, increasing the light intensity is a feasible and reliable alternative to pupil dilation to relieve the participant’s burden and to allow for performance outside the ophthalmological clinic.

## 1. Introduction

The suprachiasmatic nucleus (SCN) in the hypothalamus encompasses the endogenous biological clock, which drives physiological, endocrine, and behavioral rhythms with a periodicity of about 24 h [1]. The daily environmental light-dark cycle serves as the main synchronizer to entrain these so called circadian rhythms to an exact 24-h cycle in order to remain in phase with the 24-h period of a day on Earth [2]. Intrinsically photosensitive retinal ganglion cells (ipRGCs) play a key role in this circadian photoentrainment [3,4]. ipRGCs express the photopigment melanopsin which enables them to intrinsically decode ambient light levels [3,5]. Melanopsin is maximally responsive to short-wavelength (i.e., blue) light with peak sensitivity between 460–480 nm. In addition, ipRGCs receive extrinsic input from rods and cones [6,7]. Through direct connections between ipRGCs and the brain, the integrated intrinsic and extrinsic information on environmental light is transferred from the retina to downstream non-image forming brain regions, with the SCN as one of the main targets [8].

Besides circadian photoentrainment, ipRGCs are also involved in the regulation of mood [9], alertness [10], pineal melatonin production [11], and the pupillary light reflex (PLR) [6]. The contribution of ipRGCs to the PLR is mediated through direct projections onto the olivary pretectal nucleus (OPN; i.e., the brain region for pupil size control [12]). The involvement of ipRGCs in the PLR allows for a non-invasive method to measure functionality of the intrinsic melanopsin-based signaling pathway [13]. More specifically, the characteristic sustained contraction of the pupil after blue light can be almost entirely assigned to the melanopsin-driven delay in ipRGC repolarization after light offset [14,15]. This phenomenon, known as the post-illumination pupil response (PIPR) [8], can thus be used to estimate the functionality of the intrinsic melanopsin-signaling circuitry [14].

Accordingly, we have previously developed a reliable and robust method to assess individual differences in the maximum PIPR [16] and showed its relevance for circadian entrainment [17]. The assessment procedures include pharmacological pupil dilation in the illuminated eye [18], which is highly uncomfortable for the participants [19]. The mydriatics that are used for pupil dilation, like tropicamide, phenylephrine, and cyclopentolate, entail several possible side effects including a dry mouth, blurred vision, stinging, and sensitivity of the eyes to light [20]. In addition, dilation of the pupil may persist up until 24 h after administration causing prolonged reduced sight, which is often experienced as unpleasant and impedes tasks like driving and reading [21]. A more serious risk is that pharmacological pupil dilation may lead to closed-angle glaucoma [22]. Ophthalmological expertise is therefore indispensable for the use of mydriatics. This proficiency is usually present in eye research. However, given the myriad of ipRGC-driven downstream photoregulatory functions, PIPR assessments are also highly relevant for non-ophthalmic research on sleep [17,23,24], cognition [25], alertness [10,26], mood and emotion [27], where ophthalmologic expertise to safely apply pupil dilation may be insufficient. Taken together, to relieve the participant’s burden and to allow for a wide application of safe PIPR measurements, it would be highly desirable to have a protocol that does not rely on the use of mydriatics while preserving the high reliability and robustness of the assessments.

Previous studies have presented reliable PIPR measurements without pharmacological mydriasis [28,29]. The PIPR in these previous studies was only transient, however, while we here aimed to elicit a more melanopsin-specific maximum prolonged PIPR. Considering that the PIPR increases with increasing light intensity [30,31,32,33], the aim of the present study was to examine whether an individual’s maximum PIPR can be validly assessed without mydriatics by instead increasing the intensity of the light stimulus [14]. We, therefore, examined the within-subject between-day test-retest reliability of an alternative protocol without mydriatics with increased light intensity and compared this to the previously found very high reliability of the protocol with mydriatics [16]. In a previous different protocol to assess PIPR, outcome measures showed variability across seasons [34]. We, therefore, evaluated whether our protocol without mydriatics might result in a more stable trait-like biomarker, robust across seasons, by assessing the test-retest reliability across the winter and summer.

## 2. Materials and Methods

### 2.1. Participants

A total of 18 volunteers (5 males, mean age ± SD (range): 34.6 ± 13.6 (22–69) years) were enrolled. Volunteers were assessed at two different locations with different experimenters and the same equipment: the Netherlands Institute for Neuroscience in Amsterdam (12 participants, 5 males, mean age ± SD: 35.8 ± 15.5 years) and PsyQ Expertise Center Adult ADHD in The Hague (6 participants, 0 males, mean age ± SD: 32.3 ± 9.4 years). All participants were informed about the procedure, signed informed consent, and did not receive any incentive. None of the participants had a self-claimed history of ocular pathology and none reported to use any medication known to influence the PLR. According to Nagel anomaloscope tests, none of the participants had a color vision deficiency. The study was approved by the Medical Ethical Committee of the VU University Medical Center Amsterdam (protocol NL43319.029.13) and adhered to the tenets of the Declaration of Helsinki.

### 2.2. Pupillometry

**Figure 1:**
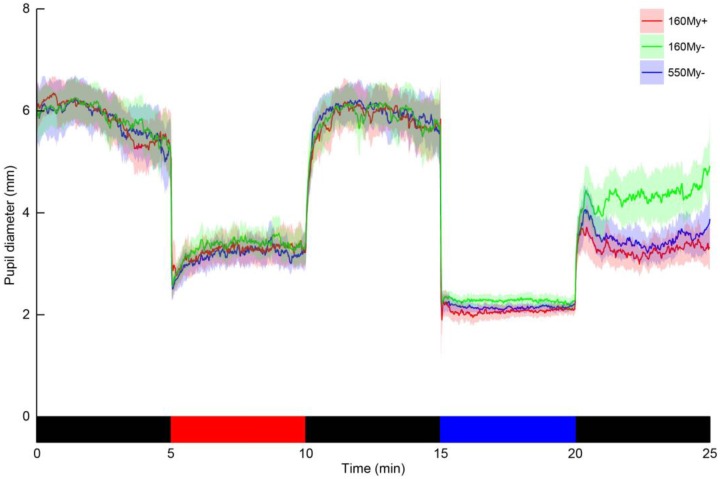
The change in pupil diameter of the left eye throughout the light exposure protocol in three different conditions. The traces represent the population mean pupil diameter, with the semi-transparent areas indicating the 95%-confidence interval, for each of the three conditions (red trace: the condition including 160 µW/cm^2^ blue light with the use of mydriatics (160My+), green trace: the condition including 160 µW/cm^2^ blue light with the pupil in its natural state (160My−), blue trace: the condition including 550 µW/cm^2^ blue light with the pupil in its natural state (550My−)). The bottom bar indicates the light exposure sequence, which was equal for all three condition (black = dark, red = monochromatic red light and blue = monochromatic blue light).

The previously established pupillometry procedures have been described elsewhere [16]. In brief, the right eye was exposed to an illumination protocol, while the left eye was continuously recorded using a custom-made infrared pupillometry set-up. The surface of the light source was 16 × 10 cm and the distance between the illuminated eye and the light stimulus was 5 cm. The light exposure protocol consisted of the following five 5-min intervals: baseline dark, monochromatic red light (635 nm) to maximize the pupil response after blue light, dark [35], monochromatic blue light (465 nm), and post-blue dark (Figure 1). These longer stimulus durations allow for more specific assessment of the melanopsin-signaling pathway because of the low sensitivity and slow kinetics of ipRGCs [3,36]. Previous research showed that light adaption of ipRGCs was completed after 5 min of light exposure, probably saturating the ipRGCs response [37]. From the baseline and post-blue pupil diameter two PIPR outcome parameters were calculated, the (1) PIPR-mm and the (2) PIPR-% [38,39]. We previously showed that these PIPR measurements have very high within-subject test-retest reliability [16].

PIPR-mm = baseline pupil diameter − post-blue pupil diameter(1)

PIPR-% = 100 * PIPR-mm/baseline pupil diameter(2)

The previously established protocol to assess the PIPR involved a blue light stimulus with an irradiance level of 160 µW/cm^2^ (Table 1) and the pupil of the right eye was dilated with the use of mydriatics, tropicamide 0.5% (160My+). The duration and intensity of the blue light stimulus [37,40], in combination with the pharmacological pupil dilation in the illuminated eye [18], were expected to saturate the melanopsin-driven photoresponse, resulting in a maximum PIPR. In order to examine whether the application of mydriatics substantially adds to eliciting the maximum PIPR this saturating PIPR assessment condition was compared to the condition with the same blue stimulus of 160 µW/cm^2^ but with the pupil in its natural undilated state (160My−). In order to assess whether increasing the intensity of the light stimulus may compensate for the possible reduced PIPR in the absence of pharmacological mydriasis the previously used saturating PIPR assessment condition was also compared to the condition in which the light intensity was increased, 550 µW/cm^2^ during blue light and the illuminated pupil was kept in its natural undilated state (550My−). In view of comments from participants in a previous pilot study, reporting major discomfort during exposure to a stimulus of such intensity with tropicamide application, we refrained from assessing the PIPR after intensified blue light with pharmacological mydriasis. Light intensities were calibrated using a spectrometer (AvaSpec-3648-USB2, Avantes, Apeldoorn, The Netherlands). The light stimuli activate all photoreceptors, the effect on each photoreceptor during each light stimulus are shown in Table 1. The melanopic lux indicates the strength of the melanopsin photoresponse. The order of the three conditions was randomized with 1 to 7 days between measurements. Full recovery of tropicamide application generally takes place within 6 h. In some cases, however the effects may last up until 24 h after administration [21]. PIPR assessments within a participant were therefore separated by at least 24 h. All sessions were performed between 9:30 a.m. and 4:00 p.m. in the months February and March 2016. To avoid time-of-day effects [41], the timing of the assessments was consistent within participants. All participants were invited to return to the lab on two consecutive days in the months July and August 2016 in order to assess the test-retest reliability of the 550My− protocol. The summer assessments were performed at the same time of day as the winter assessments. In order to assess the long-term cross-seasonal test-retest reliability between the winter and summer season, the PIPR outcome parameters of the first summer sessions were compared to the winter assessments.

### 2.3. Statistical Analysis

For both PIPR-mm and PIPR-%, differences between 160My+ and 160My− and differences between 160My+ and 550My− were visually inspected using Bland-Altman plots [43]. To evaluate whether 160My+ and 160My− procedures yield similar PIPR results a two-way random effect single measurement intraclass correlation coefficient (ICC) for absolute agreement was calculated [44]. Additional ICC values were calculated to examine reliability in pupil diameter during the baseline and post-blue intervals between 160My+ and 160My−. To compare differences in PIPR outcome measures, baseline and post-blue pupil diameter between 160My+ and 550My− the same ICC analyses were performed as in the comparison between 160My+ and 160My−. Bland-Altman plots and ICC analyses of the same outcome parameters were also used to evaluate the day-to-day test-retest reliability of 550My− protocol and to examine the seasonal effects on this condition. Data processing and analyses were conducted in R (version 3.2.1, R Foundation for Statistical Computing, Vienna, Austria), using the software packages “ICC” [45], “MethComp” [46], and “cocron” [47].

## 3. Results

The values for baseline and post-blue pupil diameter, PIPR-mm and PIPR-%, are shown in Table 2. Retinal illuminance during blue light was estimated based on the mean pupil diameter during blue light to be 18850 Td in 160My+, 1545 Td in 160My−, and 4897 Td in 550My−. Bland-Altman bias indicated low agreement between 160My+ and 160My− for the PIPR-mm and PIPR-%. In addition, there was low reliability between the PIPR outcome parameters in these conditions, as indicated by the ICC (PIPR-mm: −0.12; PIPR-%: −0.27) (Table 2). Negative ICC estimates indicate within-subject variability exceeding between-subject variability, and thus a very poor ICC [44]. There was also a low agreement between 160My+ and 160My− for the post-blue pupil diameter (ICC = 0.03).

**Figure 2:**
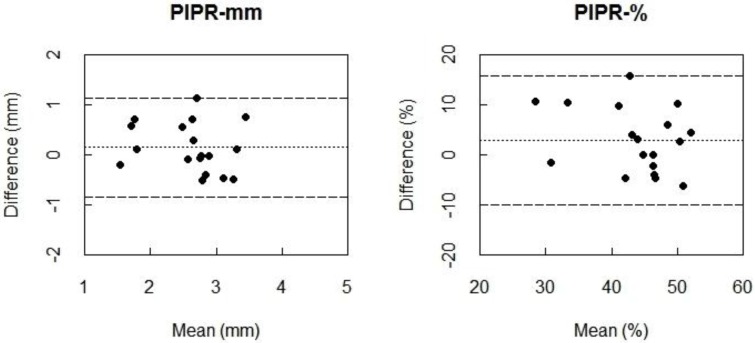
Bland-Altman plots for PIPR-mm (**left**) and PIPR-% (**right**) between 160 µW/cm^2^ blue light with mydriatics (160My+) and intensified blue light (550 µW/cm^2^) with natural pupil (550My−). Differences between the two conditions (i.e., 160My+ minus 550My−) are plotted against the mean of the two measurements. The dotted line represents the bias, i.e., the mean difference between all measurements of 160My+ and 550My−. The dashed lines are the 95% limits of agreement, 95% of the differences between the conditions lies between these lines. The smaller the limits of agreement, the better the agreement between the two measurements.

The Bland-Altman plots indicated almost zero bias between 160My+ and 550My− for PIPR-mm and PIPR-% (Figure 2) indicating high agreement. When comparing the condition 160My+ to the condition 550My−, the ICC values indicate a moderate to strong agreement for the PIPR outcome parameters (PIPR-mm: ICC = 0.67; PIPR-%: ICC = 0.58). The ICC of 0.77 indicated a strong agreement between these conditions for the post-blue pupil diameter, which was confirmed by an almost-zero Bland-Altman bias.

During baseline dark there was an almost perfect agreement between 160My+ and 160My− (ICC = 0.88) and also between 160My + and 550My− (ICC = 0.92). Bland-Altman biases also indicated this high similarity (Table 3).

**Figure 3:**
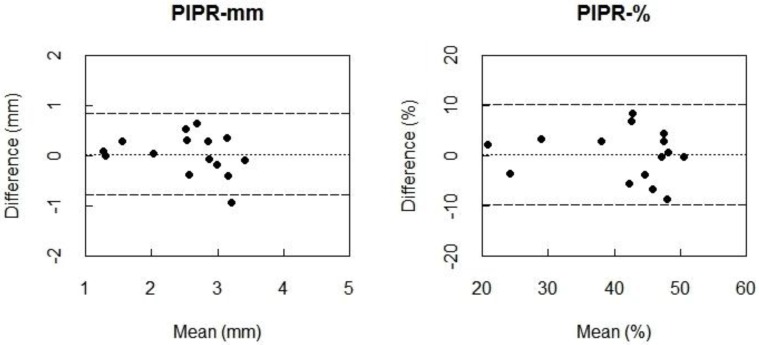
Bland-Altman plots for PIPR-mm (**left**) and PIPR-% (**right**) between the two consecutive summer assessments of the protocol with intensified blue light (550 µW/cm^2^) with a natural pupil (550My−). Differences between the two measurements (i.e., summer session 1 minus summer session 2) are plotted against the mean of the two measurements. The dotted line represents the bias, i.e., the mean difference between all measurements of the first session and all measurements of the second session. The dashed lines are the 95% limits of agreement: 95% of the differences between the conditions lies between these lines.

Fifteen (5 males, mean age ± SD (range): 36.4 ± 14.3 (23–70) years) out of the 18 participants returned to the lab for two consecutive summer assessments, one day apart. Bland-Altman plots indicated almost zero bias between the outcome measures of the 550My− protocol for PIPR-mm and PIPR-% (Figure 3), indicating high agreement. The ICC values for the PIPR outcome parameters moreover indicated a very high day-to-day test-retest reliability (PIPR-mm: ICC = 0.85; PIPR-%: ICC = 0.87) (Table 4).

**Figure 4:**
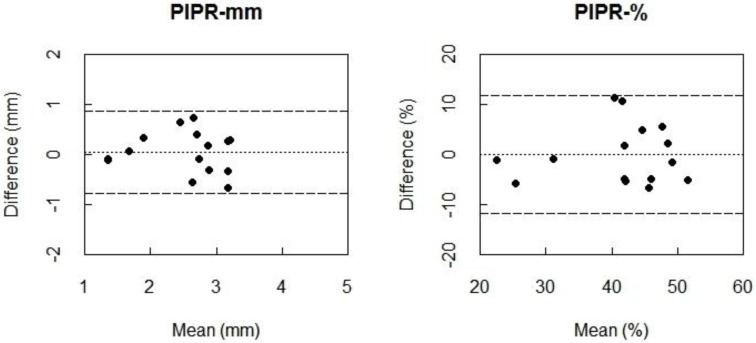
Bland-Altman plots for PIPR-mm (**left**) and PIPR-% (**right**) between the first summer sessions and winter assessments of the protocol with intensified blue light (550 µW/cm^2^) with a natural pupil (550My−). Differences between the two measurements (i.e., summer minus winter) are plotted against the mean of the two measurements. The dotted line represents the bias, i.e., the mean difference between all measurements of the summer and the winter.

The Bland-Altman plots also indicated almost zero bias between 550My− winter and 550My− summer measurements for PIPR-mm and PIPR-% (Figure 4), indicating high agreement. Again, the ICC values indicated a very high reliability for the PIPR outcome parameters (PIPR-mm: ICC = 0.83; PIPR-%: ICC = 0.80) (Table 5).

## 4. Discussion

In order to allow for a wide application of assessing the functionality of the melanopsin-signaling circuitry, we here examined whether an individual’s maximum PIPR can be validly assessed without mydriatics by instead increasing the intensity of the light exposure. In our previously established paradigm, with mydriatics, the 5-min duration of the blue light stimulus was expected to saturate the ipRGCs [16,37,40], leading to a maximum PIPR. Shorter blue light stimuli durations of milliseconds to a few seconds, as used in previous studies, may propose less discomfort for the participant but only elicit a transient PIPR (i.e., the post-exposure pupil diameter returns to baseline already during the assessment) [28,29,30,32]. On the contrary, our use of high intensity light stimuli of longer duration, tailored to the slow photoresponse and low sensitivity of ipRGCs, allows for more specific targeting of melanopsin-signaling pathway. Furthermore, in a previous shorter protocol to assess the PIPR, outcome measures showed variability across seasons [34]. Our longer protocol may result in a more trait-like biomarker that is stable across the winter and summer.

The previously established blue light stimulus with an irradiance of 160 µW/cm^2^, evoked a reduced PIPR when the pupil was kept in its natural dynamic state compared to the condition with the use of mydriatics. This indicates that dilation of the pupil or intensifying the light is required to evoke a maximum PIPR.

The retinal illuminance during blue light was over four times higher for the previously established protocol with mydriatics compared to the condition with intensified blue light without mydriatics. In spite of this fourfold difference, the PIPR did not systematically differ between these conditions. This suggests that during both conditions the ipRGCs response was saturated and a maximum PIPR was generated, since there was no further increase in PIPR even though the blue-light retinal illuminance was higher in the previously established protocol.

The moderate to strong agreement between the previously established 160 µW/cm^2^ blue light with mydriatics protocol and the alternative protocol with intensified blue light with a natural pupil indicates that both approaches are valid for eliciting the maximum PIPR. However, there can be individual variation between the results obtained with these two approaches, indicating that they should not be used interchangeably.

The within-subject test-retest of the protocol with intensified blue light with a natural pupil showed a very high agreement for both PIPR-mm and PIPR-%, approaching the reliability of our formerly established protocol with 160 µW/cm^2^ blue light and mydriatics (PIPR-mm: ICC = 0.90; PIPR-%: ICC = 0.87). This indicates that although the pupil is dynamic and the amount of light reaching the retina has a larger variation in the protocol with intensified blue light without mydriatics, this does not seem to affect the reliability of this protocol, suggesting saturation of the response at a certain intensity.

Previous research showed that the post-illumination contraction amplitude following blue light was larger in summer compared to winter [34]. However, there was only a difference between summer and winter for photopically adapted eyes. Our protocol with intensified blue light and a natural pupil showed no effect of season on the PIPR outcome parameters. We showed a very high agreement between the winter and summer measurements. Our protocol did not include an adaption period and provided the light stimulus for a much longer duration (i.e., 5 min instead of 1 s). The high reliability of our protocol with intensified blue light and a natural pupil, between days and even between seasons, provides it as a sensitive and robust possible biomarker to study trait-like individual differences in ipRGCs functioning.

In patients with retinal diseases pharmacological pupil dilation may not be without risk since dilation can lead to a rise in intraocular pressure [22]. With ipRGCs affected in diverse optic nerve and retinal diseases such as glaucoma [38,48], age-related macular degeneration [49], retinitis pigmentosa [50], and diabetes [51], PIPR measurements without dilation may provide a useful diagnostic tool. Excluding pharmacological dilation of the pupil from PIPR assessments is not only beneficial for ophthalmic patients, but also for individuals without eye-related complaints. Since pharmacological pupil dilation may be uncomfortable for participants and can even be dangerous when driving [52]. With the current reliable PIPR assessment protocol a maximum PIPR can be evoked without pupil dilation making the protocol accessible for non-ophthalmic research fields, such as sleep [17,23,24], cognition [25], alertness [10,26], mood and emotion research [27]. In addition, considering the important role ipRGCs play in circadian rhythms through their direct projections to the SCN, our current protocol can be easily applied in chronobiological studies.

A limitation of the study is the unequal gender distribution, there were more females included than males, which may have confounded our PIPR results. Accordingly, some parameters of the PLR were shown to be mildly affected by gender [53]. Future studies should, therefore, use a larger sample with a more even gender distribution to examine the effects of gender. Furthermore, future studies should examine whether the proposed protocol without pupil dilation is applicable in patients suffering from eye diseases and whether it may be used for diagnosing abnormalities in the melanopsin-signaling circuitry. 

## 5. Conclusions

The aim of the present study was to evaluate an accessible PIPR assessment protocol without mydriatics to relieve the participant’s burden and to allow for a wide application of safe PIPR measurements. This protocol was an adaption of an established reliable pupillometry paradigm, which was designed to evoke a maximum PIPR in order to estimate functionality of the intrinsic melanopsin-dependent circuitry [16]. In spite of its reliability, its reliance on mydriatics may impede widespread use of the protocol. We therefore omitted pharmacological pupil dilation and instead increased the intensity of the light stimuli. For most participants this adapted protocol, without mydriatics, showed reasonable agreement with the previous protocol, with mydriatics, in PIPR outcome measures and there was no bias between the two methods. In addition, the protocol without mydriatics showed very high test-retest reliability for the PIPR outcome parameters, both between consecutive days as well as across seasons. In conclusion, the method presented here can be used to reliably quantify trait-like individual biomarkers of the functionality of the intrinsic melanopsin-signaling circuitry.

## Figures and Tables

**Table 1:** Illuminance and luminance levels of the blue and red light during the previously established light conditions and the intensified light conditions. Since the light stimuli activate all photoreceptors, we estimated the photoinduced activation of each specific photoreceptor, expressed in α-opic lux, for each light stimulus.

Parameter		Previously Used Blue Light	Intensified Blue Light	Previously Used Red Light	Intensified Red Light
Peak wavelength (nm) (full width half maximum)		465 (20)	465 (20)	635 (20)	635 (20)
Irradiance (µW/cm^2^)		158.75	548.96	178.16	473.34
Log photon flux (1/cm^2^/s)		14.57	15.11	14.75	15.18
Illuminance (lux (V(λ))		120.91	421.13	327.03	843.35
Luminance (cd/m^2^)		375	1300	375	1000
α-opic lux					
S cone	Nsc (λ)	1131.92	3889.54	12.25	61.76
Melanopsin	Nz (λ)	1060.86	3680.33	8.21	36.84
Rod	Nr (λ)	751.94	2608.91	12.78	43.59
M cone	Nmc (λ)	378.40	1315.53	114.67	297.14
L cone	Nlc (λ)	198.81	691.60	419.58	1085.07

Details about the calculation of these values can be in found elsewhere [42].

**Table 2:** Outcome parameters of the pupil measurement from the three different conditions.

Parameter	160My+			160My−			550My−		
	Mean ± SD	Min	Max	Mean ± SD	Min	Max	Mean ± SD	Min	Max
Baseline (mm)	5.91 ± 0.81	4.04	7.22	5.97 ± 0.79	4.08	7.25	5.96 ± 0.81	4.09	7.51
Post-Blue (mm)	3.23 ± 0.53	2.20	4.35	4.33 ± 0.88	2.65	5.96	3.43 ± 0.59	2.36	4.71
PIPR-mm	2.68 ± 0.57	1.44	3.83	1.65 ± 0.80	0.51	3.30	2.54 ± 0.66	1.41	3.51
PIPR-%	45.23 ± 6.61	29.89	55.08	27.40 ± 12.34	8.22	55.49	42.27 ± 8.32	22.99	54.03

The first 2 rows represent the mean pupil diameter during middle three minutes of the 5 min interval. 160My+, 160 µW/cm^2^ blue light with mydriatics; 160My−, 160 µW/cm^2^ blue light with natural pupil; 550My−, intensified blue light (550 µW/cm^2^ blue light) with natural pupil; PIPR, Post-Illumination Pupil Response.

**Table 3:** Reliability and agreement of the baseline and post-blue pupil diameter and the two PIPR outcome parameters when comparing the previously established protocol both to the condition with the same blue light intensity with a natural pupil as well as to a condition with intensified blue light and a natural pupil.

Parameter	160My+ − 160My−		160My+ − 550My−	
	ICC (95% CI)	Bland-Altman Bias (95% Limits of Agreement)	ICC (95% CI)	Bland-Altman Bias (95% Limits of Agreement)
Baseline	0.88 (0.71–0.95)	−0.06 (−0.84 to 0.73)	0.92 (0.80–0.97)	−0.05 (−0.72 to 0.62)
Post-Blue	0.03 (−0.42–0.47)	−1.10 (−2.40 to 0.91)	0.77 (0.50–0.91)	−0.20 (−0.87 to 0.48)
PIPR-mm	−0.12 (−0.54–0.35)	1.04 (−0.54 to 2.62)	0.67 (0.32–0.86)	0.15 (−0.84 to 1.14)
PIPR-%	−0.27 (−0.64–0.21)	17.83 (−5.36 to 41.01)	0.58 (0.19–0.82)	2.96 (−9.97 to 15.89)

PIPR, Post-Illumination Pupil Response; ICC, Intraclass Correlation Coefficient; 160My+, 160 µW/cm^2^ blue light with Mydriatics; 160My−, 160 µW/cm^2^ blue light with natural pupil; 550My−, intensified blue light (550 µW/cm^2^ blue light) with natural pupil; In Bland-Altman bias the 160My+ minus 160My− and 160My+ minus 550My−.

**Table 4:** Outcome parameters of the two consecutive summer assessments of the condition with intensified blue light with a natural pupil and test-retest reliability outcomes.

Parameter	Summer Session 1 550My−			Summer Session 2 550My−			ICC (95% CI)	Bland-Altman Bias (95% Limits of Agreement)
	Mean ± SD	Min	Max	Mean ± SD	Min	Max		
PIPR-mm	2.55 ± 0.67	1.30	3.36	2.52 ± 0.77	1.23	3.67	0.85 (0.62–0.95)	0.03 (−0.79 to 0.85)
PIPR-%	41.34 ± 9.30	21.97	50.39	41.61 ± 9.85	19.69	52.28	0.87 (0.67–0.95)	0.18 (−9.84 to 10.20)

PIPR, Post-Illumination Pupil Response; ICC, Intraclass Correlation Coefficient; 550My−, intensified blue light (550 µW/cm^2^ blue light) with a natural pupil.

**Table 5:** Outcome parameters from winter and summer measurements of the condition with intensified blue light with a natural pupil and reliability outcomes from summer and winter assessments.

Parameter	Winter 550My−			Summer 550My−			ICC (95% CI)	Bland-Altman bias (95% limits of agreement)
	Mean ± SD	Min	Max	Mean ± SD	Min	Max		
PIPR-mm	2.51 ± 0.70	1.41	3.51	2.55 ± 0.67	1.30	3.36	0.83 (0.57–0.94)	0.05 (−0.78 to 0.89)
PIPR-%	41.35 ± 8.83	22.99	54.03	41.34 ± 9.30	21.97	50.39	0.80 (0.51–0.93)	−0.01 (−11.82 to 11.80)

Summer 550My−, is the first summer assessments; PIPR, Post-Illumination Pupil Response; ICC, Intraclass Correlation Coefficient; 550My−, intensified blue light (550 µW/cm^2^ blue light) with a natural pupil. The Bland-Altman bias was defined as the summer minus the winter assessment.

## Abbreviations

The following abbreviations are used in this manuscript:

SCN	Suprachiasmatic Nucleus
ipRGCs	intrinsically photosensitive Retinal Ganglion Cells
PLR	Pupillary Light Reflex
OPN	Olivary Pretectal Nucleus
PIPR	Post-Illumination Pupil Response
ICC	Intraclass Correlation Coefficient
160My+	160 µW/cm^2^ blue light Mydriatic pupil dilation
160My−	160 µW/cm^2^ blue light Natural undilated pupil
550My−	550 µW/cm^2^ blue light Natural undilated pupil
